# Rush Medical College

**Published:** 1868-10-15

**Authors:** 


### *Rush Medical College.*

*Opening of the Twenty-sixth Annual Session.* The introductory exercises of the college took place on Wednesday evening, the 30th of September. Beside an unusually large number of students, the spacious lower lecture-room was crowded with visitors. We were especially gratified at the presence of very many well-known and distinguished practitioners, both from the city and country.

After prayer by the Rev. Dr. Patterson, the Chaplain, President Blaney welcomed the class in appropriate terms, and, having given a brief sketch of the history and present position of the institution, introduced Prof. Edwin Powell, who pronounced the introductory lecture. It is unnecessary for those who know Dr. P., to say that it was a high-toned and scholarly production, replete with sound sense and elevated views — rising at times to an elegance of diction and eloquence of expression, which elicited rounds of enthusiastic applause from the audience. We had hoped to give it to the readers of the Journal in its entirety, but regret that as yet we have failed to prevail upon its author to furnish us with the manuscript.

The progress of Rush Medical College has been, since its re organization in 1859, so steadily onward —its advantages and numbers so constantly improving and increasing, that we are fain to fall back on the almost stereotyped, but strictly true, formula — its prospects were never before so brilliant as they are now. Harmony prevails in its counsels, professional zeal inspires its teachers — its spacious and commodious building is well filled with students, whom even a brief observation will convince, are above the average grade of intelligence and culture in medical schools. These things, taken in connection with the enlarged means of illustration now at its command, and the great clinical advantages it affords, will work out in the best *practical* manner the great problem of "the elevation of the profession."

We write these lines somewhat in the exultant spirit of one who has had a small part in bringing them to pass, but more as a journalist who is proud to chronicle the triumphs which painstaking effort and zealous industry will ever, in the end, attain over noisy declamation, witless diatribes, and unscientific, loose and impractical experiments in "Medical Reform."

